# Anticandidal Activity of Omiganan and Its Retro Analog Alone and in Combination with Fluconazole

**DOI:** 10.1007/s12602-021-09757-9

**Published:** 2021-03-02

**Authors:** Paulina Czechowicz, Maciej Jaśkiewicz, Damian Neubauer, Grażyna Gościniak, Wojciech Kamysz

**Affiliations:** 1 Department of Microbiology, Faculty of Medicine, Wroclaw Medical University, Wroclaw, Poland; 2 Department of Inorganic Chemistry, Faculty of Pharmacy, Medical University of Gdańsk, Gdansk, Poland

**Keywords:** Antimicrobial peptides, Biofilm, *Candida albicans*, Fluconazole, Omiganan, Retro analogs, Vulvovaginal candidiasis

## Abstract

**Abstract:**

Vulvovaginal candidiasis (VVC) is a vaginal infection that manifests itself as several symptoms which can lead to various life-threatening complications. The majority of VVC is caused by *Candida albicans* strains, and it is estimated that approximately 75% of women worldwide would suffer from this condition at least once during their lifetime. Surprisingly, the detailed pathomechanism of yeast-like fungi invasions in vagina is not yet fully understood. However, the ability to form biofilm on vaginal mucosa is considered as one of the critical factors associated with failure of the therapy and recurrences of the disease. Antimicrobial peptides (AMPs) are a promising class of compounds that are receiving a growing interest owing to their antibacterial, antifungal, and antibiofilm properties. Omiganan is a synthetic analog of Indolicidin that is characterized by wide spectrum of antimicrobial and antibiofilm activities*.* Recent reports suggest improved activity of analogs with a reversed sequence (retro-analog concept). Therefore, Omiganan and its retro analog were tested against planktonic forms and biofilm of 18 *Candida* strains isolated from VVC. Moreover, the synergy between the AMPs and fluconazole was studied as well. The AMPs appeared to be effective against *C. albicans* biofilm, and the reversion of the sequence generally led to an improved antimicrobial activity. Furthermore, confocal and scanning electron microscopic visualizations revealed the effectiveness of AMPs-fluconazole combinations also against fluconazole-resistant strains.

**Graphical Abstract:**

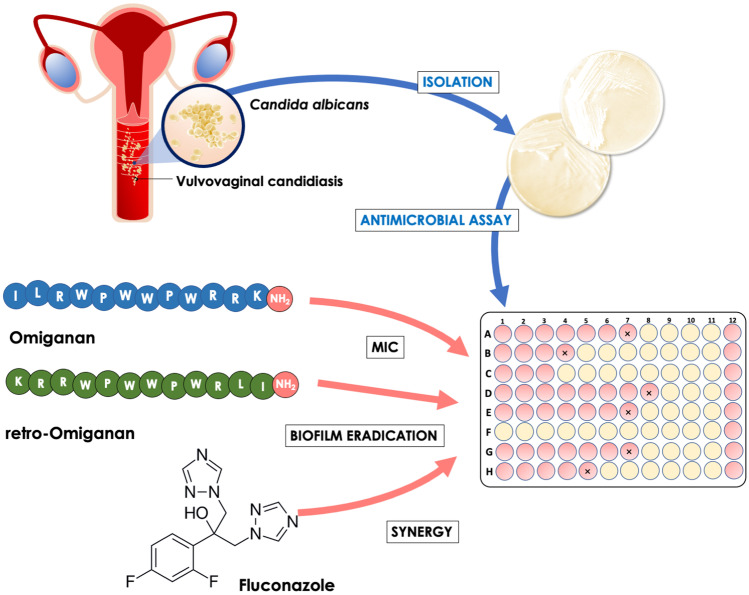

## Introduction

*Candida* species are considered to be one of the commonest etiological factors of vaginal and cervical infections. It is estimated that approximately 75% of women in childbearing age would suffer from a symptomatic episode of vulvovaginal candidiasis (VVC) at least once during their lifetime, even if any predisposing factors had not occurred [1–4]. Moreover, the typical recurrent VVC defined as four or more episodes per year, develop in about 10% cases of VVC, but in general, relapses occur in about half of the patients [3–6]. At the same time, approximately 20% of women are asymptomatic carriers of *Candida* strains in the vagina [3, 7]. Although the virulence factors of yeast-like fungi are quite well known, a detailed pathomechanism of vulvovaginal candidiasis still remain unclear [8]. Moreover, the role of potential factors enabling colonization and invasion of *Candida*, as well as specific recurrence tendencies, remain also unexplained [9]. Many authors suggest that the ability to form biofilm is essential for development of vaginal infections [2, 7, 10]. Meanwhile, the number of multidrug-resistant *Candida* strains has increased and conventional antifungals such as fluconazole (FLC) began to be inefficient. This particularly applies to fungi from the NCAC group (*Non-C. albicans Candida*) like *C. glabrata* and *C. krusei.* For this reason, methods of alternative therapy are constantly being sought. Among the compounds which are worth mentioning are those synthesized by bacteria, which are characterized by probiotic as well as antimicrobial activity, also against *Candida*. Among the microorganisms with probiotic anti-*Candida* potential, there are mainly bacteria of the genus *Lactobacillus*, including *L. acidophilus*, *L. rhamnosum*, *L. crispatus*, *L. fermentum*, and *L. brevis* [11–16]. Besides their probiotic potential, these strains are characterized by production of variety antimicrobial compounds like organic acids, ethanol, enzymes, and hydrogen peroxide andantimicrobial peptides (AMPs) [17, 18]. The last one mentioned belongs to the promising and large group of compounds being a part of innate immune system of almost all organisms [19]. Apart from their wide-range of antimicrobial and immunomodulatory properties, some AMPs are characterized by unique antifungal as well as anti-biofilm activity [20]. Among these, a prominent representative is Omiganan, which is a synthetic analog of indolicidin - a peptide isolated from the cytoplasmic granules of bovine neutrophils [21]. This compound is characterized by a wide spectrum of antimicrobial activity, and it is one of AMPs that has lately passed the III phase of clinical trials [22]. To date, several studies reported possibility to use Omiganan against yeast-like fungi with promising results [21, 23–27]. However, there is a lack of research focused on its antibiofilm activity. In addition, recent studies indicate that its retro-analog (with reversed sequence) showed an improved activity against bacteria and fungi [28, 29]. On the basis of this development, it has been postulated that both compounds (Omiganan and retro-Omiganan) could effectively be used in the treatment of *Candida* infections associated with biofilm. Moreover, there are few studies that indicate synergistic anti-*Candida* effect of combination of azoles (e.g., fluconazole) with various AMPs [30–35]. This effect could probably be associated with different mechanisms of action of both groups of compounds. Regrettably, to date, Omiganan has not been investigated in this regard so far. The aim of this study was to learn whether or not Omiganan and its retro analog could be effective against planktonic forms and biofilm of clinical strains of *C. albicans* isolated from women with VVC. In addition, synergy with fluconazole has been tested.

## Materials and Methods

### Peptide Synthesis

Omiganan and retro-Omiganan (sequences in Table 1) were synthesized manually by solid-phase method using Fmoc chemistry on polystyrene Rink amide resin (Orpegen Peptide Chemicals GmbH, Heidelberg, Germany). Deprotection of the Fmoc group was performed with 20% (v/v) piperidine (Merck, Darmstadt, Germany) solution in DMF (Honeywell, Seelze, Germany) for 15 min. Acylation was conducted in a DCM/DMF(1:1, v/v) solution with coupling reagents Fmoc-AA-OH, OxymaPure, and DIC (mole ratio 1:1) for 1.5 h using a threefold molar excess. Every step was preceded by rinsing the resin and running the chloranil test. Peptides were cleaved from the resin using a mixture of TFA (Apollo Scientific, Denton, UK), TIS (Sigma-Aldrich, St. Louise, MO, USA), phenol (Sigma-Aldrich, St. Louise, MO, USA), and deionized water (92.5:2.5:2.5:2.5, v/v/v/v). The cleavage was accomplished for 1.5 h with agitation. The peptides were purified by reversed-phase high-performance liquid chromatography, and their identity was confirmed by mass spectrometry (ESI-MS).

**Table 1:** Peptides used in this study

Name	Sequence	Net charge	Average mass (Da)	MS analysis		
				z	m/z calc	m/z found
Omiganan	ILRWPWWPWRRK-NH_2_	+ 5	1779.15	2 3 4	890.6 594.1 445.8	890.3 594.0 445.8
retro-Omiganan	KRRWPWWPWRLI-NH_2_			2 3 4	890.6 594.1 445.8	890.5 594.1 445.9

### Candida Strains and Microbiological Assay

Microbiological assays were performed on 18 clinical strains of *C. albicans* acquired from the Internal Collection of the Department of Microbiology, Wroclaw Medical University. All analyzed strains were once originally isolated from vagina and cervix of women suffering from a fungal infection. Reference strain of *C. albicans* ATCC 10231 was obtained from the Polish Collection of Microorganisms (PCM, Polish Academy of Sciences, Wroclaw).

In the first step, Minimum Inhibitory Concentrations (MICs) of fluconazole (Merck, KGaA, Darmstadt, Germany), Omiganan, and retro-Omiganan were determined on 96-well polystyrene plates following microdilution method described by Clinical and Laboratory Standards Institute [36]. For this purpose, 24-h cultures of *C. albicans* from Sabouraud Dextrose Agar with chloramphenicol (100 mg/L) were transferred to sterile PBS (AppliChem GmBH, Darmstadt, Germany) to achieve a density of 0.5 McFarland (1–5 × 10^6^ cells per mL). Then the suspension was diluted 1:1000 in RPMI 1640 medium (Merck, KGaA, Darmstadt, Germany). Working solutions of AMPs and fluconazole were prepared by dissolution in PBS and DMSO (Merck, KGaA, Darmstadt, Germany), respectively. Subsequently, the range of concentrations (0.5–256 µg/mL) of test compounds was prepared and the inoculums were added (1–5 × 10^3^ cells per mL). All plates prepared in this way were then incubated for 24 h at 37˚C. For peptides, the MIC value was defined as a lowest concentration at which a noticeable growth of fungi was inhibited. For fluconazole, it was the concentration that inhibited at least 50% of yeast growth. Cell densities were determined spectrophotometrically at 530 nm (BiochromAsys UVM 340 Microplate Spectrophotometer, Biochrom Ltd, USA). Yeast growth was calculated based on the measured optical density (OD) at 530 nm using the following equation: (OD_well_ − OD_background_)/(OD_growth_control_ − OD_background_) × 100%. The procedure was conducted in triplicate and included growth and a sterility control.

### Activity Against Biofilm

Minimum biofilm eradication concentrations (MBECs) were determined on 96-well polystyrene flat bottom plates with resazurin (7-hydroxy-3H-phenoxazin-3-one 10-oxide) as a cell viability reagent [37]. For this purpose, the 0.5 McFarland inoculum was diluted 1:100 (final concentration 1–5 × 10^4^ cells per mL) and 100 µL of that suspension was added into the test plates. After 24 h of incubation at 37 °C, the wells of the plates were rinsed three times with phosphate buffer saline (PBS) to remove non-adherent cells. Subsequently, 100 µL of the test compounds over a concentration range of 0.5–256 µg/mL were added to each well. After 24 h of incubation at 37 °C, 20 µL of the resazurin solution (4 mg/mL) was added. The MBEC values were read out after 1 h of incubation at 37 °C with shaking (120 rpm). The values were recorded as the lowest concentration at which the reduction of resazurin (from blue to pink) was lower or equal to 10 ± 0.5% as compared to the positive (100%) and negative (0%) controls. All experiments were performed in triplicate and included growth and a sterility control.

### Fractional Inhibitory Concentration Index

To study interactions between fluconazole and the peptides, fractional inhibitory concentration (FIC) index was determined, following the checkerboard method [38]. FIC index was calculated for the reference strain of *C. albicans* ATCC 10231 and for two randomly selected clinical strains by the lowest (number 18.) and the highest (number 13.) MIC values for fluconazole. For this purpose, serial dilutions of antimicrobial agents were prepared as previously for MIC determination. Horizontal wells were applied for fluconazole, while vertical ones for test of AMPs and the final concentrations range was 0.5–64 µg/mL. Subsequently, the inoculum of the test strain was added, and the plates were incubated for 24 h at 37 °C. Inhibition of fungal growth was assessed visually. The following formula was used to calculate the FIC index:

$\frac{A}{\mathrm{MICA}}$ + $\frac{B}{\mathrm{MICB}}$ = FIC_A_ + FIC_B_ = FICindex,

averageΣFIC = $$\frac{\sum \mathrm{FIC}}{n}$$

where *A* and *B* are inhibitory concentrations of the agents determined by the checkerboard method. MIC values were determined previously (individual compounds) and FIC_*A*_ and FIC_*B*_ were the ratio of the two values; *n* is the number of FIC indices. The FIC index values were interpreted according to EUCAST guidelines [39]. The results are shown in Table 2. The experiments were performed in duplicate for each strain.

**Table 2:** Correlation between FIC and the effect of the combination of antimicrobial agents

FIC index	Effect
≤ 0.5	Synergy
> 0.5 to 1.0	Additive
> 1.0 to ≤ 2.0	Indifference
> 2.0	Antagonism

### Microscopic Assay

Visualization of the results was carried out for selected strains, namely, those nos. 13 and 18 using confocal microscopy and scanning electron microscopy (SEM). The microscopy was carried out in the Laboratory of Electron Microscopy of the University of Life Sciences in Wroclaw.

### Confocal Microscopy

Samples of biofilms of both selected strains (nos. 13 and 18) and the reference strain of *C. albicans* ATCC 10231 were prepared directly on coverslips. Culture preparation and biofilm growth were carried out in the same way as that described for MBEC determination. For this purpose, the 24-h mature biofilms were exposed to fluconazole, Omiganan and retro-Omiganan at MBEC concentrations and two combinations of fluconazole with Omiganan or retro-Omiganan. Concentrations of combinations of the compounds were selected based on FIC values with 8 µg/mL of fluconazole and 4 µg/mL of each peptide. For this combination FIC values did not exceed 0.5 and can be interpreted as synergy of both compounds. After 24-h incubation at 37 °C, the LIVE/DEAD *BacLight* Bacterial Viability Kit (Thermo Fisher Scientific, Waltham, MA, USA) was added to stain biofilms for 30 min in the dark. SYTO™ 9 (excitation wavelength 488 nm) was taken up by live fungal cells (green), while propidium iodide (excitation wavelength 532 nm) stained only dead cells (red). Negative control involved coverslips that were exposed to UV lamp for 30 min. All samples were viewed under a LeicaDMi8 confocal microscope at a 630 × magnification.

### Scanning Electron Microscopy

Biofilm samples prepared in 8-well plates were fixed with 2.5% glutaraldehyde (Argenta, Poland). Further cell fixation was followed by incubation in 10% formalin (Argenta) and dehydration in increasing concentrations (20, 40, 60, 80, 95, and 100%) of ethanol (Argenta) for 1 min each step. Finally, the samples were incubatedfor 1 min in acetone (Argenta). The dried samples were affixed to SEM stubs and placed into a gold/palladium sputter coater. The specimens were viewed at × 1500–5000 magnification in a ZEISS EVOL515 scanning electron microscope.

## Results

### Minimum Inhibitory Concentration

All tested compounds exhibited an antimicrobial activity against reference and clinical strains of *C. albicans* (Table 3).

**Table 3:** MIC values (μg/mL) of fluconazole, Omiganan and its retro analog against clinical strains of *C. albicans* isolated from VVC and reference *C. albicans* ATCC 10231

Strain no	MIC (µg/mL)		
	Fluconazole	Omiganan	Retro-Omiganan
1	0.5	128	32
2	0.125	64	32
3	0.125	128	32
4	0.25	128	64
5	0.25	128	32
6	0.125	64	32
7	0.125	128	64
8	16	64	32
9	0.125	128	32
10	0.125	64	32
11	0.125	64	32
12	0.25	64	32
13	256	64	32
14	0.125	64	32
15	4	256	128
16	0.125	128	32
17	0.125	128	64
18	0.125	64	32
*C. albicans* ATCC 10231	1	128	32

**Fig. 1:**
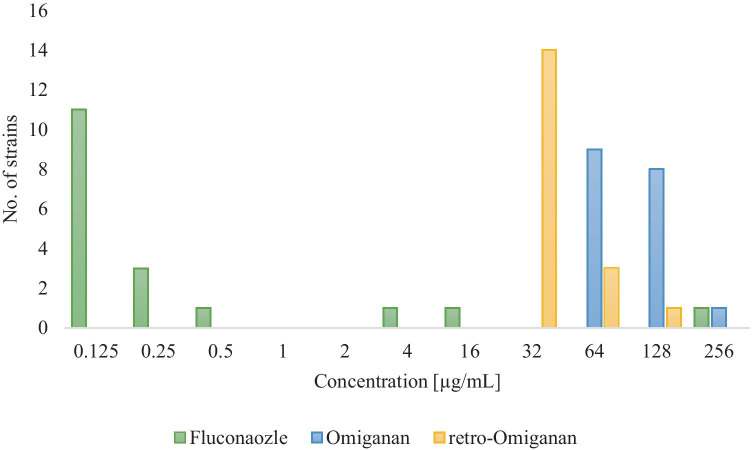
MIC values distribution of fluconazole and the AMPs

MIC values of fluconazole for the vast majority of the tested strains did not exceed 4 µg/mL (except strain nos. 8 and 13), and the commonest value was 0.125 µg/mL. Moreover, only one *C. albicans* strain appeared to be highly resistant with a MIC value above 64 µg/mL. Interestingly, strains less sensitive to fluconazole were more susceptible to peptides. MIC value distribution of the compounds against clinical strains of *C. albicans* strains is presented in Fig. 1.

MIC values of Omiganan ranged from 64 to 256 µg/mL, while the concentration of 64 µg/mL was the commonest value (9/18 strains). In the case of retro-Omiganan, MIC values ranged between 32 and 128 µg/mL, one being that of 32 µg/mL (in 14 strains out of 18 tested). To evaluate peptides selectivity, the results of our previous study on cytotoxicity against HaCaT cell line (immortalized human keratinocytes) were included in calculations of SI indices (Table 4) [29]. The geometric mean (GM) of the MIC values against *C. albicans* was calculated. SI is the IC_50_ to GM ratio.

**Table 4:** IC50, GM, and selectivity indices (SI) of the AMPs determined for clinical strains of *C. albicans*

Peptide	GM	IC_50_ Jaśkiewicz et al. [29]	SI
Omiganan	94.06	79.39	0.84
Retro-Omiganan	38.79	29.51	0.76

### Minimum Biofilm Eradication Concentration

In Table 5, minimum biofilm eradication concentration (MBEC) values of fluconazole and the tested peptides against reference and clinical *Candida* strains are presented.

**Table 5:** MBEC values (μg/mL) of the fluconazole, Omiganan and its retro analog against clinical strains of *C. albicans* isolated from VVC and reference *C.* *albicans* ATCC 10231. GM determined for clinical strains is included

Strain no	MBEC (µg/mL)		
	Fluconazole	Omiganan	Retro-Omiganan
1	512	256	64
2	512	256	128
3	512	256	128
4	512	256	256
5	512	256	128
6	512	256	64
7	512	512	128
8	256	256	64
9	512	256	128
10	512	256	128
11	512	256	64
12	512	128	64
13	512	256	64
14	512	256	128
15	512	256	128
16	512	256	128
17	512	256	128
18	512	256	64
GM	492.66	256.00	101.59
*C. albicans* ATCC 10231	512	256	64

**Fig. 2:**
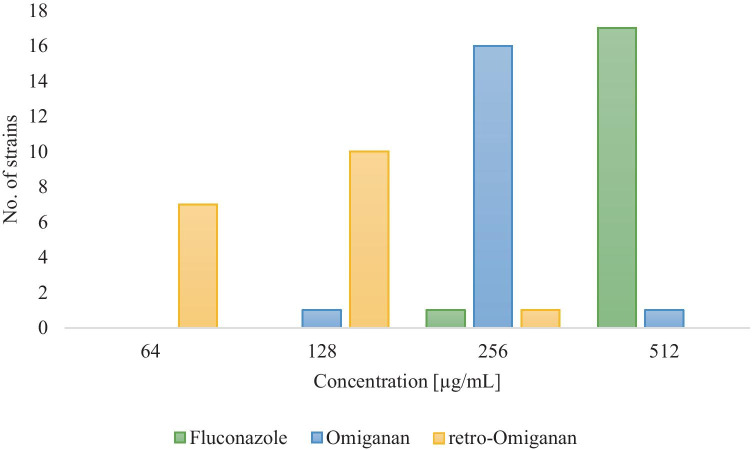
MBEC distribution of fluconazole and tested AMPs

MBEC values of the azole attained a top value of 512 µg/mL, while MBEC ones of the two AMPs were at most twice as high as the corresponding MIC value. MBEC distribution of fluconazole and peptides against that of clinical *C.* *albicans* strains used in this study is displayed in Fig. 2.

The MBEC values of Omiganan ranged from 128 to 512 µg/mL and most often were 256 µg/mL (for 16 of 18 strains). With retro-Omiganan MBEC values ranged between 64 and 256 µg/mL, with the top one of 128 µg/mL (10/18 strains).

### FIC Index

The FIC index values reveal an additive effect of AMPs and fluconazole combinations (FICi 0.5–1) against planktonic forms of *C. albicans* in concentrations up to 8 times lower than when using each of the compounds separately. The FICi for Omiganan and fluconazole slightly exceeded 0.5 for both the reference strain ATCC 10231 and the *C. albicans* strain which was resistant to fluconazole (no. 13), reaching a value of 0.578. Interestingly, for the randomly selected strain susceptible to fluconazole (no. 18), FIC index of 4.125 indicated antagonism. FIC indices of retro-Omiganan combined with fluconazole were 0.780 and 0.687 for *C. albicans* ATCC 10231 and FLC-resistant strain, respectively. Similarly, FIC index for the susceptible to azole strain was 4.133 indicating antagonism of both compounds as well (Table 6).

**Table 6:** FIC index values of combination of fluconazole with Omiganan and retro-Omiganan against *C. albicans* ATCC 10231 reference strain and two clinical *C. albicans* strains isolated from VVC

	Omiganan + fluconazole	retro-Omiganan + fluconazole
*C. albicans* ATCC 10231	0.578	0.780
Strain no. 13	0.578	0.687
Strain no. 18	4.125	4.133

**Fig. 3:**
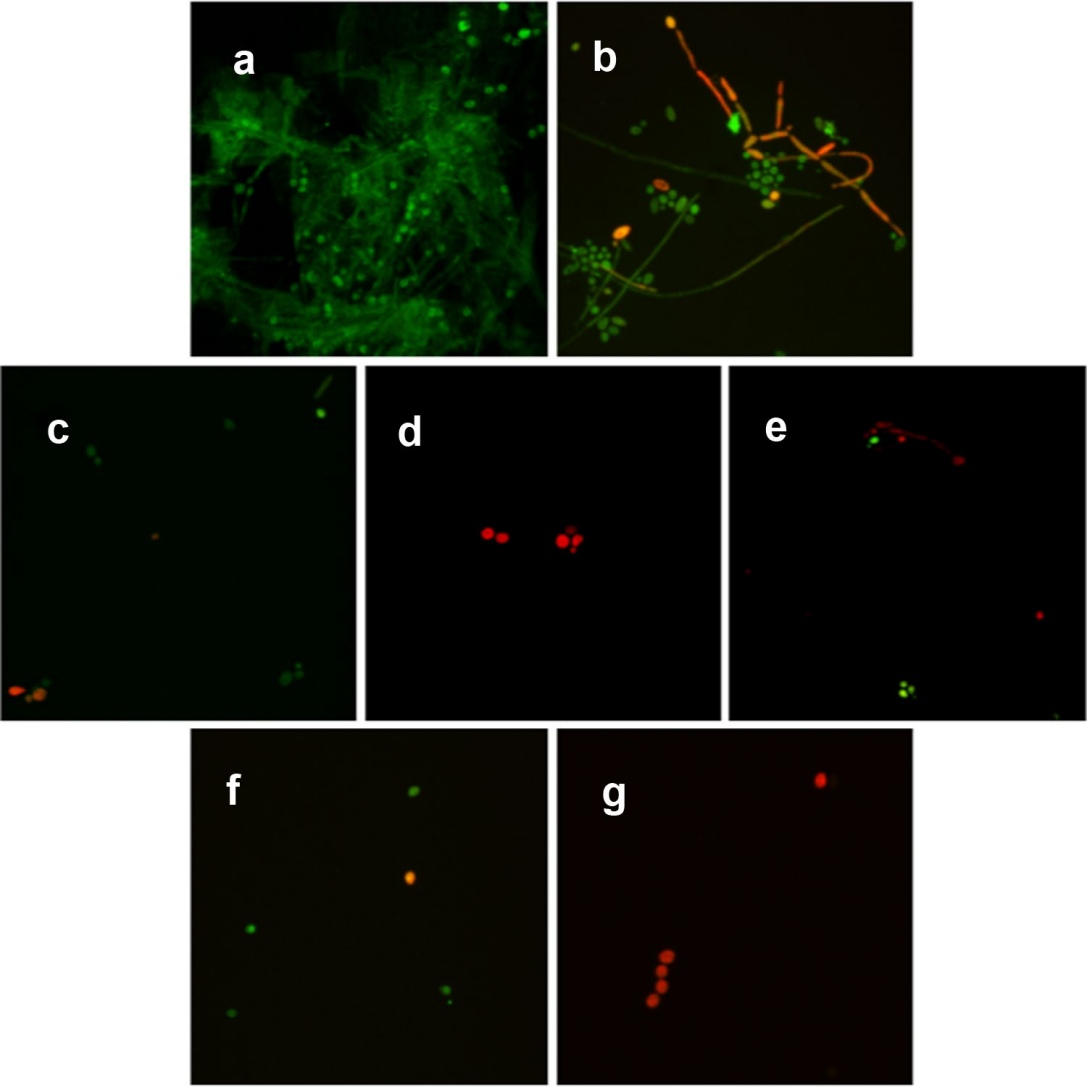
Confocal fluorescence microscopy images of *Candida* biofilm. **a** Untreated biofilm, and biofilm treated with **b** UV radiation, **c** fluconazole, **d** Omiganan, **e** Retro-Omiganan, **f** combination of fluconazole and Omiganan, and **g** combination of fluconazole and retro-Omiganan

Confocal fluorescence micrographs of the AMPs and fluconazole treated *Candida* biofilm are presented in Fig. 3.

In confocal microscopy, the biomass of *Candida* biofilm treated with test compounds in concentrations equal to the respective MBEC and FIC values (8 µg/mL of fluconazole and 4 µg/mL of each peptide) was almost completely eradicated. Uptake of PI (red fluorescence) is the result of the increased membrane permeability indicating cell death. Figure 3 a. and b represent positive (untreated biofilm) and negative control (biofilm exposed to UV radiation) respectively. Evidently biofilm consists of blastospores and elongated filaments. On micrographs 3.C., 3.D., and 3.E. there are biofilms treated with the compounds at MBEC concentrations (fluconazole, Omiganan, retro-Omiganan, respectively). Reduction in biofilm biomass is seen, but among few surviving cells, the majority is recognized as dead blastospores (stained red). Similar effect was noticed with combinations. In this case, almost all biofilms were eradicated, and the remaining cells were mostly dead. Surprisingly, confocal microscopy revealed that combinations of Omiganan and retro-Omiganan with fluconazole were also effective in strain no. 18 for which FICi value indicated antagonism.

**Fig. 4:**
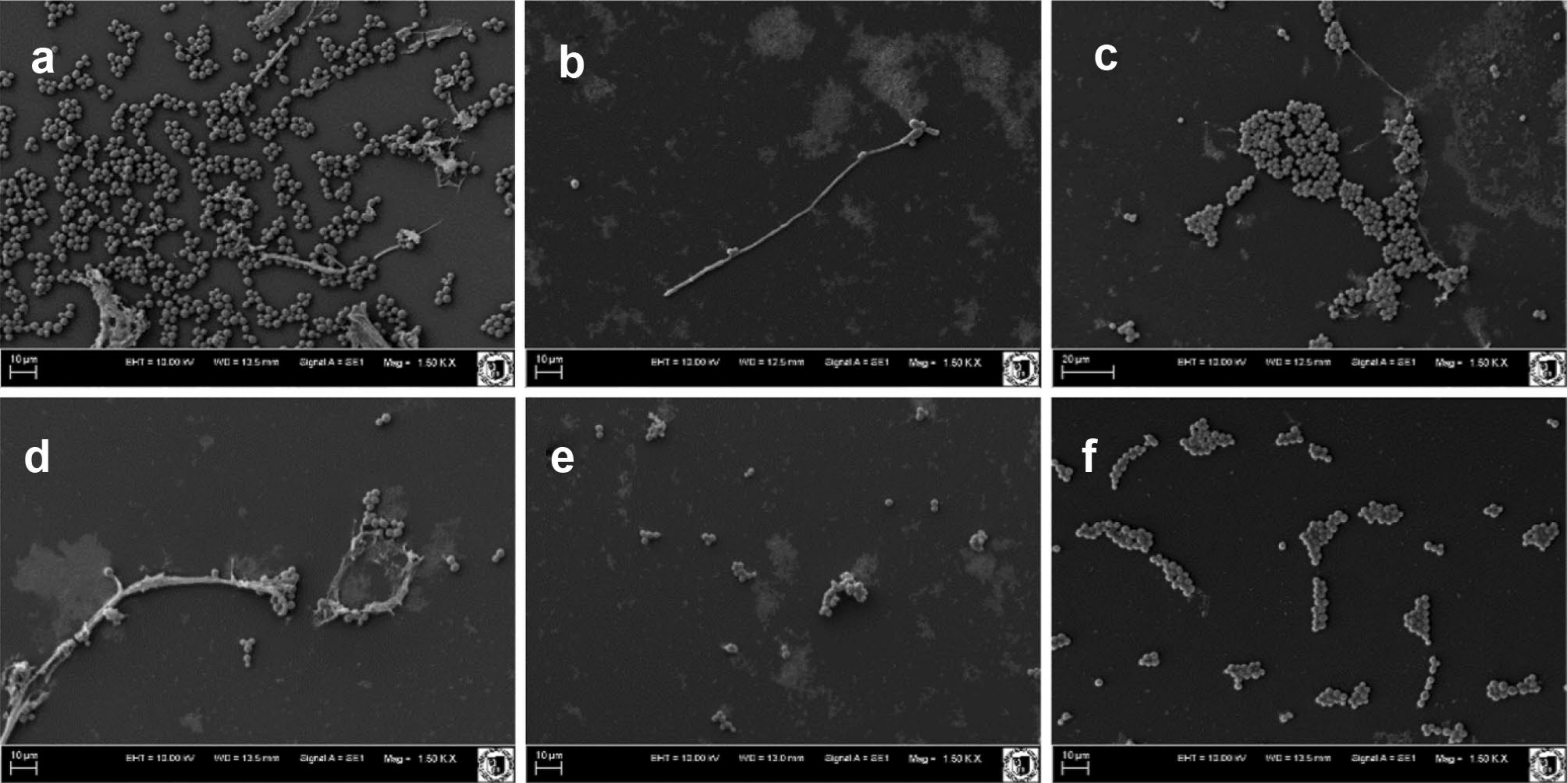
Scanning electron microscopy images of *Candida* biofilm - **a** Untreated biofilm, and biofilm treated with **b** Fluconazole, **c** Omiganan, **d** Retro-Omiganan, **e** combination of fluconazole and Omiganan, and **f** Combination of fluconazole and retro-Omiganan

Scanning electron microscopy has also shown that, apart from the reduction of biofilm biomass, blastospores are seen among the few surviving cells in the presence of the tested compounds (in MBEC and FIC concentrations) and no filamentation occurs (Fig. 4). Figure 4 a represents a positive control (untreated biofilm) in which multiple blastospores clusters and single hyphae are noticed. Treatment with MBEC concentrations of fluconazole and retro-Omiganan (4.B. and 4.D.) resulted in reduction of biofilm with single filaments and few blastospores surviving. Unfortunately, Omiganan at MBEC concentration failed to eradicate biofilm (Fig. 4c) as indicated by blastospores remaining in clusters. Concentrations equal to the FIC values (8 µg/mL of fluconazole and 4 µg/mL of each peptide) were almost as effective in biofilm eradication as MBEC values with reduction in biofilm biomass with no filamentation.

## Discussion

Vulvovaginal candidiasis remains the second commonest gynecological infection in women worldwide, significantly reducing their life quality and comfort [3, 40, 41]. Although an increasing percentage of VVC infections is caused by species belonging to the NCAC group, which are resistant to the classic treatment regimen, *C. albicans* remains the major etiologic factor of fungal vaginitis [2, 5, 7, 10]. It was speculated that therapeutic failures during the empirical use of azoles - mainly fluconazole - were associated with an increasing resistance of *C. albicans* strains against this group of antimycotics. Meanwhile, vaginal yeasts that were isolated in clinical practice tend to be susceptible to fluconazole [1, 5, 42–44]. This fact may provide an argument to the hypothesis that the formation of a highly resistant and impermeable biofilm on the vaginal mucosa by *Candida* species is responsible for the lack of therapeutic effect of using azoles [2, 5, 7, 8, 10, 40, 41]. Hence, intensive search for alternative therapies in the treatment of fungal infections (including VVC) focuses, among others, on testing compounds that can potentially be active against biofilm. For instance, AMPs are the compounds with antimicrobial and antibiofilm potential and Omiganan is one of the extensively studied [29, 45]. A recently published report on enhancement of antimicrobial activity of selected AMPs indicate that reversion of Omiganan sequence results in enhanced activity against microorganisms with a slight contribution of hemolysis [28]. Accordingly, the aim of this study was to determine, whether or not the effect would have been observed in the case of clinical strains of *Candida* isolated from VVC. For this purpose, evaluation of the antimicrobial activity was conducted against both the planktonic form and biofilm. The obtained MIC values for Omiganan and its retro analog against 18 clinical and one reference strain of *C. albicans* ATCC 10231 confirmed the hypothesis [28]. Moreover, the amino acid sequence reversion resulted in a distinctly increased antifungal activity. Geometric mean of MIC values of Omiganan was substantially higher (94.06) than that of the retro analog (38.79). A similar effect was achieved with regard to the biofilm structure formed by the strains. MBEC values of both AMPs were twice and four times as high as the corresponding MIC values. GMs of MBEC values for Omiganan and retro-Omiganan were 256.00 and 101.59, respectively. Considering MIC values and IC_50_ against HaCaT cell line determined by our group in the previous study [28], it seemed worthwhile to estimate selectivity indices (Table 4). Those for Omiganan and its retro analog are similar amounting to 0.84 and 0.76, respectively. Interesting results were obtained during a pilot study on the effect of the fluconazole-peptide combination against *C. albicans*. For both, the reference strain and the only fluconazole-resistant strain among the tested strains, the FICi values indicate either a synergistic or an additive effect of the combination of azole with Omiganan or its retro analog - in concentrations many times lower than the corresponding MIC values. Meanwhile, with the strain susceptible to fluconazole, the result was opposite, indicating antagonism of both azole-AMP pairs. Despite this small group of the analyzed strains, it was decided to verify the results using confocal microscopy and scanning electron microscopy. Microscopic analyses confirmed the finding that using combinations of fluconazole with peptides at concentrations determined by the checkerboard assay resulted in eradication of fungal biofilm. Few cells remaining in the field of view were dead (as visualized by LIVE/DEAD staining) and filamentation did not appear (Figs. 3 and 4). Perhaps this phenomenon is caused by different mechanisms of action of both groups of compounds. It is well known that molecular target of fluconazole is Erg11 enzyme (14α-lanosterol demethylase), which is involved in the synthesis of ergosterol. Under the influence of fluconazole, a change occurs in the composition of the cell membrane, its liquefaction and an increase in permeability to K^+^ and ATP ions, which results in a fungistatic effect [46]. In contrast, Omiganan and retro-Omiganan interact directly with the cell membrane leading to its permeabilization and cell death. Hypothetically, the permeabilization of membranes by AMPs can support the penetration of fluconazole into its intracellular molecular target. Fluconazole also affects the structure of the cell membrane, thus facilitating its permeabilization by AMPs. Similar, although not yet understood, synergistic or additive effects of fluconazole with various AMPs against *Candida spp.* strains (especially FLC-resistant fungi) were reported [30–35]. It was claimed that combination of different compounds as described above could be one of the most promising approach as to develop alternative therapy against multidrug resistant or biofilm-forming yeast-like fungi. The results obtained in this study remain only a highly optimistic and promising premise and may constitute a starting point for further research on the effects of both Omiganan and its retro analog on *Candida* yeast-like fungi. Due to the limited number of the strains tested, the results require verification on a larger pool of *Candida* isolates, which also includes strains from the NCAC group. In the course of further work, it would undoubtedly be worthwhile to determine the toxicity of Omiganan and its retro analog to human cell lines. Cytotoxicity studies should include compounds (fluconazole and peptides) alone and combined (according to FICi values) to learn how specific combinations with synergistic activity determined in this study affect normal human cells. Cytotoxicity of AMPs seems to be a serious drawback and further studies on the proposed combination of antimicrobial agents can shed the light on this important issue. Beneficial effect can be expected owing to a considerably lower effective concentration of AMPs when combined with fluconazole.

## Conclusions

Preliminary results of our study, as the first or one of first research of this kind, indicate antimicrobial activity of Omiganan and its newly synthesized retro analog against clinical strains of yeast-like fungi *Candida* isolated from women with VVC. Despite a small pool of investigated strains, there is a tendency indicating higher anticandidal activity of retro-Omiganan than the parent peptide - both against planktonic cells as well as biofilm. The promising results were noticed in the case of synergy studies. For both compounds, a potential synergistic effect in combination with conventionally used fluconazole was indicated. However, there is a need to confirm our findings in further studies on a wider range of clinical strains. This may lead for application of a well-known antimycotic and peptides in combination therapy in concentrations much lower than when used separately - non-toxic to eukaryotic cells, and therefore safer.

## Data Availability

All data generated or analyzed during this study are included in this published article.

## Abbreviations

ACN: Acetonitrile
AMPs: Antimicrobial peptides
ATCC: American type culture collection
CLSI: Clinical and Laboratory Standards Institute
DCM: Dichloromethane
DIC: *N,N′-*Diisopropylcarbodiimide
DMF: *N,N*-Dimethylformamide
DMSO: Dimethyl sulfoxide
ESI–MS: Electrospray-ionization mass spectrometry
FIC: Fractional inhibitory concentration
FICi: Fractional inhibitory concentration index
FLC: Fluconazole
Fmoc: 9-Fluorenylmethoxycarbonyl group
MBEC: Minimum biofilm eradication concentration
MIC: Minimum inhibitory concentration
PBS: Phosphate-buffer saline
TFA: Trifluoroacetic acid
TIS: Triisopropylsilane
VVC: Vulvovaginal candidiasis
